# Learning a Foreign Language: A Review on Recent Findings About Its Effect on the Enhancement of Cognitive Functions Among Healthy Older Individuals

**DOI:** 10.3389/fnhum.2018.00305

**Published:** 2018-07-30

**Authors:** Blanka Klimova

**Affiliations:** Department of Applied Linguistics, Faculty of Informatics and Management, University of Hradec Králové, Hradec Králové, Czechia

**Keywords:** healthy older adults, foreign language learning, cognitive functioning, efficacy, benefits, a lack of evidence

## Abstract

Currently, there is an increasing number of older population groups, especially in developed countries. This demographic trend, however, may cause serious problems, such as an increase in aging diseases, one of which is dementia whose main symptom consists in the decline of cognitive functioning. Although there has been ongoing pharmacological research on this neurological disorder, it has not brought satisfying results as far as its treatment is concerned. Therefore, governments all over the world are trying to develop alternative, non-pharmacological strategies/activities, which could help to prevent this cognitive decline while this aging population is still healthy in order to reduce future economic and social burden. One of the non-pharmacological approaches, which may enhance cognitive abilities and protect against the decline in healthy older population, seems to be the learning of a foreign language. The purpose of this mini-review article is to discuss recent findings about the effect of foreign language learning on the enhancement of cognitive functions among healthy older individuals. The findings, divided into three research areas, show that the learning of a foreign language may generate a lot of benefits for older individuals, such as enhancement of cognitive functioning, their self-esteem, increased opportunities of socializing, or reduction of costs. However, as Ware et al. (2017) indicate, any intervention program on foreign language learning should be well thought of and tailored to the needs of older people in order to be effective and avoid accompanying factors, such as older people’s anxiety or low self-confidence. Nevertheless, more empirical studies should be done in this area.

## Introduction

The population is aging. For example, in Europe, older people aged 65+ years form 18% of the whole population. It is expected that by 2050, the older population will outnumber the young population in many developed countries (Statista, 2017). This demographic trend, however, may cause serious problems, such as an increase in aging diseases, one of which is dementia whose main symptom consists in the decline of cognitive functioning. This is connected with the brain atrophy, particularly in the temporal cortex, the region that is related to declarative memory (see Buckner, 2004), which is encoded by the hippocampus, entorhinal cortex and perirhinal cortex, loss of synaptic connections (Maston, 2010), and the occurrence of neuropathological symptoms associated with dementia (see Antoniou and Wright, 2017). Although there has been ongoing pharmacological research on this neurological disorder, it has not brought satisfying results as far as its treatment is concerned (Karakaya et al., 2013).

Therefore, governments all over the world are trying to develop alternative, non-pharmacological strategies/activities, which could help to prevent this cognitive decline while this aging population is still healthy in order to reduce future economic and social burden (Maresova et al., 2016). These alternative, non-pharmacological intervention therapies can be divided into several groups, which have a positive impact on the enhancement of cognitive functions: physical activities, cognitive training, healthy diet (see Klimova and Kuca, 2015), as well as social enhancement interventions (see Ballesteros et al., 2015), including the use of modern information and communication technologies (Peter et al., 2013; Ballesteros et al., 2014). One of the cognitive training activities, which may enhance cognitive abilities and protect against the decline in healthy older population, seems to be the learning of a foreign language (see Antoniou et al., 2013; Kroll and Dussias, 2017). As Connor (2016) points out, learning a foreign language can promote thinking skills, increase mental agility and delay the aging of the brain. However, as Kurdziel et al. (2017) explain, the retrieval of new words among older people is harder since their fluid intelligence (i.e., the ability to reason and solve things), as well as the working, short-term, memory (i.e., management of immediately available information) are getting affected in the course of aging. On the contrary, their crystallized intelligence (i.e., the ability to use experience, knowledge and skills) remain intact in the aging process (see Kavé et al., 2008). Kurdziel et al. (2017) also state that the decline in language ability among older people is slower than the decline in global memory. In addition, older individuals possess a superior raw vocabulary even if compared with well-educated adults of young generation. In addition, foreign language learning does not have any side effect (Bak, 2016) and can help reduce country’s economic burden (Bialystok et al., 2016). Simply, it does not do any harm (see Strauss, 2015). Abutalebi and Clahsen (2015) present that knowledge about language processing in older individuals and about the potential factors that prevent cognitive decline is currently very much desirable since it may contribute to preparing for the demographic changes which our society faces.

The purpose of this mini-review article is to discuss recent findings about the effect of foreign language learning on the enhancement of cognitive functions among healthy older individuals.

## Methods

The methodology of this mini-review article is based on Moher et al. (2009). Studies were selected on the basis of the following keyword collocations: *healthy aging* and *foreign language learning*; *healthy older individuals* and *foreign language learning*, *healthy older individuals* and *bilingualism*, found in the world’s acknowledged databases: Web of Science, PubMed, Scopus and ScienceDirect. The search was not limited by time since the studies on the research topic were scarce. Altogether 43 studies, including both review and original articles, were detected, most of them were identified in ScienceDirect and Web of Science, followed by PubMed and Scopus. The analysis was done by identifying the key words and checking duplication of available sources in the databases mentioned above. Afterwards, the studies were assessed for their relevancy, i.e., verification on the basis of abstracts whether the selected study corresponds to the set goal. After the exclusion of such studies, 26 studies remained for the full-text analysis. Out of 26 studies, 12 were empirical or randomized control studies, which are in detail described in Table 1. The review studies (e.g., Antoniou et al., 2013; Lee and Tzeng, 2016; Kurdziel et al., 2017), the studies dealing with the younger adults (e.g., Schlegel et al., 2012; Bellander et al., 2016) and the studies with patients suffering from dementia, respectively Alzheimer’s disease (e.g., Woumans et al., 2015; Bialystok et al., 2016) were used for comparison reasons. Moreover, the author also explored websites connected with the research topic, e.g., SeniorsMatter (2017).

**Table 1:** An overview of the detected empirical studies on the effect of foreign language learning on the enhancement of cognitive functions among healthy older individuals.

Study	Objective	Number of subjects	Main outcome measures	Results
Ansaldo et al. (2015)	To examine the behavioral and neural traces of nonverbal interference control in healthy older bilinguals and monolinguals.	20 subjects, mean age: 74 years.	Language assessment, neuropsychological tests, magnetic resonance imaging (fMRI) scanning.	Elderly bilinguals deal with interference control without recruiting a circuit that is particularly vulnerable to aging.
Bak et al. (2014)	To explore the effect of bilingualism on later-life cognition controlling for childhood intelligence.	853 participants.	First tested in 1947 (age 11) and then at the age of 70; a series of cognitive tests for participants including intelligence test and comparing the results with their own test scores at the age of 11.	Bilinguals, as well as those who acquired a second language at the later age, performed significantly better than predicted from their baseline cognitive abilities, with strongest effects on general intelligence and reading; the findings also suggest a positive effect of bilingualism on later-life cognition, including in those who acquired their second language in adulthood.
Bak et al. (2016)	To investigate the impact of a short intensive language course on attentional functions.	67 participants at the age of 18–78 years.	Auditory tests of attentional inhibition and switching.	Even a short period of intensive language learning can modulate attentional functions and that all age groups can benefit from this effect.
Diaz-Orueta et al. (2012)	To examine and define the user requirements for developing digital learning games for older Europeans.	86 subjects at the age of 60+ years from Spain, Netherlands and Greece.	Focus group sessions with audio and video recordings.	The main aspects of interest were challenge, socialization, fun, providing learning opportunities and escape from daily routine. In addition, the content of these games should focus on foreign language learning, physical activity, or culture.
Kousaie and Phillips (2017)	To investigate the benefit of bilingualism among healthy older bilinguals and monolinguals with the help of behavioral and electrophysiological measures.	43 healthy elderly, aged between 60 years and 83 years.	Montreal Cognitive assessment, EEG recording.	There is evidence that older bilinguals execute enhanced cognitive processing than older monolingual individuals.
Lawton et al. (2015)	To explore if the age of clinically diagnosed Alzheimer’s disease and vascular dementia occurred later for bilingual than monolingual, immigrant and U.S. born, Hispanic Americans.	1789 community-dwelling Hispanic Americans, aged ≥60 years.	Cognitive testing; clinical examination; self-report using a three-point Likert-type scale for the evaluation of language proficiency.	Mean age of dementia diagnosis was not significantly different for bi/monolingual, U.S. born or immigrant, Hispanic Americans.
Ramos et al. (2017)	To explore the relationship between language learning and switching ability in elderly monolingual participants who learned a second language during a whole academic year.	43 older individuals at the age of 60–80 years.	A color-shape switching task.	The acquisition of a second language in the elderly does not necessarily lead to an enhancement of switching ability as measured by switching costs.
Sanders et al. (2012)	To verify whether non-native English speakers (n-NES) have lower risk of incident dementia/AD and that educational level might modify this relationship.	1944 healthy older individuals ≥70 years.	Battery of cognitive performance tests at baseline and each successive annual evaluation; and nested Cox proportional hazards models were used.	n-NES status does not appear to have an independent protective effect against incident dementia/AD, and that n-NES status may contribute to risk of dementia in an education-dependent manner.
Ware et al. (2017)	To determine whether the English training program integrating technology is feasible for older French people.	14 older people, average age: 75 years.	Standardized tests for measuring cognitive functions, questionnaires, post-intervention, semi-directive interviews, and a content/theme analysis.	The program was stimulating and enjoyable and it might be used as a therapeutic and cognitive intervention in future.
Wilson et al. (2015)	To test the hypothesis that foreign language and music instruction in early life are associated with lower incidence of mild cognitive impairment (MCI) and slower rate of cognitive decline in old age.	964 healthy older individuals.	Cognitive testing and clinical classification of MCI.	Higher levels of foreign language and music instruction during childhood and adolescence are associated in old age with lower risk of developing MCI but not with the rate of cognitive decline.
Yeung et al. (2014)	To determine whether bilingualism is associated with dementia in cross-sectional or prospective analyses of older adults.	1616 community-living healthy older adults.	Self-reports; cognitive testing; and clinical examination.	There is no association between speaking more than one language and dementia.
Zahodne et al. (2014)	To test the hypothesis that dementia is diagnosed at older ages in bilinguals compared to monolinguals.	1067 healthy older Hispanic immigrants in New York.	Self-report using a four-point Likert-type scale for the evaluation of language proficiency; Selective Reminding Test; Boston Naming Test; tests of verbal and nonverbal abstraction and letter fluency; Color Trails Test; and Cox regression.	There is not a protective effect of bilingualism on age-related cognitive decline or the development of dementia.

## Findings and Their Discussion

As it has been stated in the “Methods” section, there is a lack of studies on the learning of a foreign language and its effect on the enhancement of cognitive functioning in older people, apart from those on bilingualism (see Klimova et al., 2017a). Overall, the identified studies can be divided into three main areas: studies concerning the brain plasticity in the old age and foreign language learning; studies focused on foreign language learning among healthy older individuals; and studies aimed at bilingualism and healthy aging, including the electrophysiological studies. All of them also discuss the cognitive aspects.

### Plasticity of the Brain in the Old Age and Foreign Language Learning

The brain remains with considerable plasticity even in the old age. Although there is some neural deterioration that rises with age, the brain has the capacity to increase neural activity and develop neural scaffolding to regulate cognitive function (Park and Reuter-Lorenz, 2009; Reuter-Lorenz and Park, 2014). For example, Cheng et al. (2015) maintain that both short-term and long-term period of foreign language learning may lead to the changes in the structure of the brain, which consequently may contribute to the promotion of the cognitive reserve, i.e., the resilience to neuropathological damage of the brain (Stern, 2013). This has been also confirmed by Lee and Tzeng (2016), who claim that foreign language learning results in effective structural as well as functional connectivity in the brain due to neural plasticity. They indicate that the effective connectivity due to foreign language learning enhances the capacity for language processing and general executive control by reorganizing neural circuitries. Furthermore, research shows that foreign language learning has a positive impact on both white and gray matter structures (see Bellander et al., 2016). For instance, Schlegel et al. (2012) in their randomized controlled study with 11 English speakers (average age of 20 years) who took a 9-month intensive course in written and spoken Modern Standard Chinese and 16 controls who did not study a language reported that the plasticity of the white matter played a significant role in adult language learning. Although their adult learners showed progressive changes in white matter tracts, associated with traditional left hemisphere language areas and their right hemisphere analogs, the most important changes appeared in frontal lobe tracts crossing the genu of the corpus callosum-a region, which is not generally involved in current neural models of language processing. Tyler et al. (2010) in their study on preserved syntactic processing across the life span, argue that this is caused by the shift from a primarily left hemisphere frontotemporal system to a bilateral functional language network. In addition, Connor (2016) described a study of retired people doing an intensive language course of 5 h a day on the Isle of Skye to learn Gaelic (see Bak et al., 2016). After finishing the course, the scientists discovered these people were more mentally agile than those doing a course on something else. As Antoniou et al. (2013) indicate, foreign language training may engage a larger brain network than other forms of cognitive training that have been investigated (e.g., math and crossword puzzles), and it is likely to require long distance neural connections. However, not all the findings on the plasticity o the brain and aging process are positive. For instance, the controlled study by Ramos et al. (2017) maintains that the switching ability (i.e., the ability to shift attention between one task and another) was not enhanced by learning a foreign language, in this case Basque language, among elderly Spanish people.

### Foreign Language Learning Among Healthy Older Individuals

In the most recent study on foreign language learning and its effect on cognitive functioning, Ware et al. (2017) developed a technology-based English training program for older French adults. The program was based on the assumptions provided by Antoniou et al. (2013). These assumptions involved various factors, such as that computer-based language training can be administered anywhere and at any time to suit learner’s needs, the content can be adjusted and items can be repeated. In addition, learners can socialize. The average age of the participants was 75 years. The course lasted for 4 months and consisted of 16 2-h sessions. The researchers used standardized tests for measuring cognitive functions (Montreal Cognitive Assessment), as well as University of California Loneliness Assessment for measuring subjective feelings of loneliness and social isolation, both of which did not significantly change after finishing the course. Nevertheless, the researchers found out that their program was feasible for this age group and the participants enjoyed it. Similarly, research performed by Bak et al. (2016) on a short 1-week Scottish Gaelic course on attentional functions among 67 adults aged between 18 years and 78 years reveals that even a short period of intensive language learning can modulate attentional functions and that all age groups can benefit from this effect. The results showed that at the beginning there was no difference between the groups. However, at the end of the course, a considerable improvement in attention switching was detected in the language group (*p* < 0.001) but not the control group (*p* = 0.127), independent of the age of subjects. In addition, they also suggested that these short-term effects could be maintained through continuous practice, but the minimum study period should be 5 h a week.

Research also indicates that the age in second language acquisition is not such a significant factor, but the length of exposure to the target language is important (Bialystok, 1997). In fact, on the one hand, it might take older people longer and more practice to learn a foreign language in the old age because of difficulty distinguishing new sounds and retrieve novel words, but on the other hand, they are more relaxed and motivated to learn (see SeniorsMatter, 2017). As it has been already pointed out, the main problem for older people is to retrieve new words (see Kurdziel et al., 2017). However, they are able to retain these new words easily if they are provided in the context. Kurdziel et al. (2017) also revealed that newly learned words were stored in hippocampus during encoding and then integrated into lexicon in the course of sleeping. Nevertheless, the quality of sleeping is often negatively affected in the old age and therefore older people are not able to retain as many words as their younger counterparts whose sleeping period is higher and unbroken.

Diaz-Orueta et al. (2012) report that the main stimulation for older people to learn a foreign language is a challenge, socialization, fun, providing learning opportunities and escape from daily routine. Moreover, the older individuals might also have experience of learning a foreign language, which can help them in acquiring a new language (see Singleton and Lengyel, 1995).

Kurdziel et al. (2017) expand by suggesting that learning throughout aging should be a must because older people who keep mentally and physically active are less likely to be cognitively impaired and depressed. In fact, depression seems to be one of the most serious comorbidities in the aging process (Popa-Wagner et al., 2014; Sandu et al., 2015). Furthermore, foreign language learning increases self-confidence, enables older people travel and communicate with their peers in foreign countries.

### Bilingualism and Healthy Aging

The theory of bilingualism states that people acquiring a second language in their adulthood may prevent cognitive decline in later life by approximately 4.5 years (see Bialystok et al., 2007, 2016; Bak et al., 2014; Wilson et al., 2015; Woumans et al., 2015). In their recent study, on the basis of measures of cognitive function and brain structure, Bialystok et al. (2016) show that bilingualism can delay cognitive decline. As Bialystok et al. (2004) and Bialystok (2006) state, bilingualism contributes to compensate age-related losses in certain executive processes. Furthermore, bilingual people possess better mental flexibility because they are used to adapting to constant changes and processing information in a more effective way than the monolingual individuals. However, these results especially concern the retrospective studies on bilingualism since the prospective studies on bilingualism, such as Lawton et al. (2015), Sanders et al. (2012), Yeung et al. (2014), or Zahodne et al. (2014), have not exerted significant results in this respect (see Klimova et al., 2017a). For instance, Mukadam et al. (2017) in the most recent study revealed that retrospective studies inclined to confounding by education, or cultural differences in presentation to dementia and are thus not relevant to set causative links between risk factors and results. However, the electrophysiological studies on bilingualism indicate that bilingualism may enhance cognitive functions among healthy older individuals (i.e., Kousaie and Phillips, 2017). Moreover, as Ansaldo et al. (2015) state, healthy older bilinguals deal with interference control without recruiting a circuit that is particularly vulnerable to aging.

Table 1 below then summarizes the main findings of the studies on the effect of foreign language learning on the enhancement of cognitive functions for healthy older individuals.

The limitations of this mini-review study mainly involve a lack of relevant studies on the research topic. This fact may cause the overestimated effects of the results, which may have an adverse impact on the validity of these reviewed studies (see Melby-Lervåg and Hulme, 2016).

## Conclusion

Overall, some of the findings in Table 1, as well as from other mentioned studies indicate that the learning of a foreign language may generate benefits for older individuals, such as enhancement of cognitive functioning (Bak et al., 2014, 2016; Ansaldo et al., 2015; Kousaie and Phillips, 2017) their self-esteem (Ware et al., 2017), or increased opportunities of socializing (Diaz-Orueta et al., 2012; Ballesteros et al., 2015). Bialystok et al. (2016) also emphasize that second-language learning has long-term implications for public health in terms of cost-effectiveness. In addition, as Ware et al. (2017) indicate, any intervention program on foreign language learning should be well thought of and tailored to the needs of older people in order to be effective and avoid accompanying factors, such as older people’s anxiety or low self-confidence.

In comparison with the intervention studies focusing on physical activities (see Klimova et al., 2017b), there is still smaller evidence of the effect of foreign language learning on the enhancement of cognitive functions among the healthy aging population. This is especially caused by a lack of research in this area.

## Author Contributions

BK drafted, analyzed, wrote and read the whole manuscript herself.

## Conflict of Interest Statement

The author declares that the research was conducted in the absence of any commercial or financial relationships that could be construed as a potential conflict of interest.
